# Flax–Glass Fiber Reinforced Hybrid Composites Exposed to a Salt-Fog/Dry Cycle: A Simplified Approach to Predict Their Performance Recovery

**DOI:** 10.3390/polym15112542

**Published:** 2023-05-31

**Authors:** Luigi Calabrese, Dionisio Badagliacco, Carmelo Sanfilippo, Vincenzo Fiore

**Affiliations:** 1 Department of Engineering, University of Messina, Contrada Di Dio (Sant’Agata), 98166 Messina, Italy; lcalabrese@unime.it; 2 Department of Engineering, University of Palermo, Viale delle Scienze, Edificio 6, 90128 Palermo, Italy; dionisio.badagliacco@unipa.it (D.B.); carmelo.sanfilippo01@unipa.it (C.S.)

**Keywords:** hybrid composites, flax fibers, environmental degradation, mechanical properties, moisture sorption, forecasting modeling

## Abstract

Despite natural fibers gaining significant attention in recent decades, their limited performance and poor durability under humid environments cannot allow them to fully replace their synthetic counterparts as reinforcement for structural composites. In such a context, this paper aims to investigate how exposure to a humid/dry cycle affects the mechanical response of epoxy laminates reinforced with flax and glass fibers. In particular, the main goal is to assess the performance evolution of a glass–flax hybridized stacking sequence in comparison with the full glass and flax fiber reinforced composites. To this end, the investigated composites were first exposed to salt-fog for 15 or 30 days and then to dry conditions (i.e., 50% R.H. and 23 °C) for up to 21 days. The presence of glass fibers in the stacking sequence significantly stabilizes the mechanical performance of composites during the humid/dry cycle. Indeed, hybridization of inner flax laminae with outer glass ones, acting as a protective shield, hinders the composite degradation due to the humid phase also promoting performance recovery during the dry phase. Hence, this work showed that a tailored hybridization of natural fibers with glass fibers represents a suitable approach to extend the service-life of natural fiber reinforced composites exposed to discontinuous humid conditions, thus allowing their employment in practical indoor and outdoor applications. Finally, a simplified theoretical pseudo-second-order model that aimed to forecast the performance recovery shown by composites was proposed and experimentally validated, highlighting good agreement with the experimental data.

## 1. Introduction

The use of natural fibers in several industrial applications has gained significant attention in recent years due to their positive features such as low cost, light weight, and biodegradability, thus making them a potential option for the development of sustainable fiber reinforced composites [1,2]. Nevertheless, one of the main drawbacks of these materials, which hinders their broader use, is poor resistance to moisture or wet environments, which can cause the material degradation and loss of mechanical performance over time [3,4]. These issues represent the biggest bottleneck promoting the application of natural fiber reinforced composites (NFRCs), thus limiting their use in outdoor applications or in marine environments, where exposure to water or moisture is expected. In this context, hybridization with synthetic fibers such as glass fibers can improve the overall performances of NFRCs [5,6], thus extending their service life.

Indeed, glass fibers play a shielding role, preventing both moisture and water penetration into the natural fibers and hindering the degradation of the polymeric matrix [7]. In this context, Liu et al. [8] demonstrated that the addition of glass fibers can improve the aging resistance of NFRCs in severe environmental conditions, also showing that the stacking sequence of hybrid composites noticeably influenced their water absorption. Analogously, the hybridization of flax fiber reinforced composites by using basalt fibers allows to contain the mechanical properties reduction due to aging, thus improving the overall durability of the composites [9,10]. Wang et al. [11] studied the hydrothermal durability of carbon–flax hybrid composites, showing that the presence of carbon fibers on the surface leads to better hydrothermal resistance than other hybrid stacking sequences, thanks to the barrier effect of carbon fibers against water molecules. Cheour et al. [12] analyzed the effect of the stacking sequence on the durability of flax–glass fiber reinforced epoxy hybrid composites subjected to long-term immersion in water at room temperature. In particular, they monitored the water absorption of the composites along their mechanical and damping properties, showing that the hybrid composite having two inner flax layers and two outer glass layers is the most efficient for a specification where damping and bending modulus are the main criteria. The low-velocity impact response of flax–glass fiber reinforced vinyl ester composites immersed in seawater at 25 °C and 70 °C was compared with that of full glass and full flax composites by Paturel and Dhakal [13]. Another work focused on the aging resistance of flax–glass fiber reinforced polypropylene composites under water immersion at 85 °C and UV exposure [14]. A further investigation was recently addressed to assess the flexural behavior of wood beams strengthened by flax–glass fiber reinforced epoxy composites exposed to hydrothermal (50 °C and 95% RH) and weathering (i.e., cyclic water spray-UV radiation) conditions [6]. All these papers clearly indicate hybridization as a valid strategy toward achieving improved structural performance of natural fiber reinforced composites exposed to aggressive environmental conditions.

In this context, one of the most significant challenges in composite materials’ development consists in the assessment of their performance recovery at the end of exposure to humid or wet environments. Indeed, the use of composites for outdoor structural applications often involves their exposure to alternated environmental conditions, indicating temperature, humidity, and salt spray as key factors in the material degradation. For these reasons, we recently focused our attention on the behavior of composites under alternated salt-fog exposure and dry phases, thus assessing the ability of flax [15,16], glass [17], and flax–glass [18] fiber reinforced epoxy composites in the recovery of their mechanical properties under discontinuous exposure to aggressive environmental conditions, typical of marine applications. The improvement of knowledge on alternate humid/dry or wet/dry conditions opens new challenges for the use of NFRCs in outdoor or marine applications, where the exposure to discontinuous humid or wet conditions represents a critical issue. Hence, the tailoring of sustainable materials with improved durability is a relevant topic to widen their potential for use on external structures; i.e., hybridization with glass fibers could potentially represent a step toward this perspective. Furthermore, this opens up a widespread range of applications in all industrial sectors for which the choice of more environmentally sustainable materials represents an added value, without compromising its mechanical stability and durability over time.

In this context, a simplified approach aiming to predict the performance degradation and recovery of hybrid composites when exposed to discontinuous humid or wet conditions could provide valuable scientific soundness of these materials, in addition to having significant implications for their use in various application fields. This approach would also make it possible to extend this strategy not only for composite design but also for maintenance monitoring in real operating conditions.

To this end, the aging behavior of flax, glass, and flax–glass fiber reinforced hybrid composites was compared by exposing them to a salt-fog/dry cycle and monitoring their water adsorption and desorption properties along with their flexural performance. An interpretative analytical model of the kinetics of the recovery process based on the pseudo-second-order equation was also proposed, showing a good agreement between the experimental and the numerical results.

## 2. Materials and Methods

A vacuum infusion technique was employed as the manufacturing method of the investigated composite panels (30 × 30 cm^2^), by using a two-stage vacuum pump model VE 235 D (Eurovacuum, Reeuwijk, The Netherlands). Each composite was cured at 25 ± 1 °C for 24 h and post-cured at 50 ± 1 °C for 15 h, as suggested by the supplier (Mates Italiana s.r.l., Milan, Italy) of the commercial epoxy matrix (SX8 EVO). A 2 × 2 twill weave woven flax fabric (318 g/m^2^ areal weight) supplied by Lineo (Saint Martin du Tilleul, France) and a plain weave woven glass fabric (200 g/m^2^) supplied by Mike Compositi (Milan, Italy) were used as reinforcement.

Table 1 reports the reference code, the stacking sequence, the nominal thickness, the fiber, and the void content for each investigated composite. In particular, the HC, GC, and FC codes refer to the hybrid flax–glass, full glass, and full flax fiber reinforced composites, respectively.

### Aging Conditions and Mechanical Characterization

A climatic chamber SC/KWT 450 (Weiss, Buchen, Germany) was used to expose the investigated composites to salt-fog spray conditions for 15 and 30 days, respectively. During this phase, named “humid”, the temperature inside the chamber was set to 35 °C ± 1 °C, and 5 wt.% sodium chloride solution was used to fill the reservoir of the chamber, thus obtaining the salt-fog, in accordance with the ASTM B 117 standard. It is worth noting that an entire panel for each stacking sequence was exposed to the salt-fog to limit the water diffusion through the edges. At the end of the salt-fog exposure, each panel was cut by means of a diamond blade saw to obtain prismatic samples (i.e., 13 mm × 64 mm) for the mechanical characterization. In particular, five samples for each stacking sequence and each salt-fog exposure time (i.e., 15 and 30 days) were immediately tested in order to evaluate the effect of the humid phase on the mechanical properties of the investigated composites. Further samples (i.e., 35 for each composite) were stored at 50% ± 10% R.H. and 23 °C ± 2 °C in a climate-controlled room. This “dry” phase, having a duration of up to 21 days, was carried out according to the ISO 291:2008 standard. Five samples for each stacking sequence were tested, varying the dry time (i.e., after 0.5, 1, 2, 3, 7, 11, and 21 days).

For each investigated condition, three-point bending tests were performed by using a U.T.M. model Z005 by Zwick-Roell (Ulm, Germany) equipped with 5 kN load cell. The support span and the crosshead speed were set equal to 54 mm and 1.4 mm/min, according to the ASTM D790 standard. The flexural test can be identified as the best loading configuration option in order to evaluate the suitability of using the proposed hybrid stacking sequence (i.e., having flax fabric as reinforcement of the internal laminae) as an alternative of the full glass laminate.

Depending on the different aging conditions (i.e., salt-fog exposure and subsequent dry times), all the investigated batches were codified using a prefix (i.e., FC-, HC-, or GC-) related to the stacking sequence of the composite (see Table 1) followed by a suffix (i.e., HtwDtd) indicating the interval times of humid (tw) and dry (td) phases, respectively. For instance, the FC-H15D21 code refers to the flax fiber reinforced samples aged in the salt-fog chamber for 15 days and then dried for 21 days. Regardless of the stacking sequence, the H0D0 code is used to indicate the unaged samples (i.e., reference).

## 3. Results and Discussion

### 3.1. Water Adsorption and Desorption

The weight change (WC) shown by the investigated composites was calculated as a function of time during the humid/dry cycle, according to the following equation:

$$WC\left[\%\right]=100\cdot \frac{M_{ti}-M_{U}}{M_{U}}$$

where *M_U_* and *M_TI_* are the weight of unaged and aged samples at time ti (t_humid_ + t_dry_), respectively.

**Figure 1:**
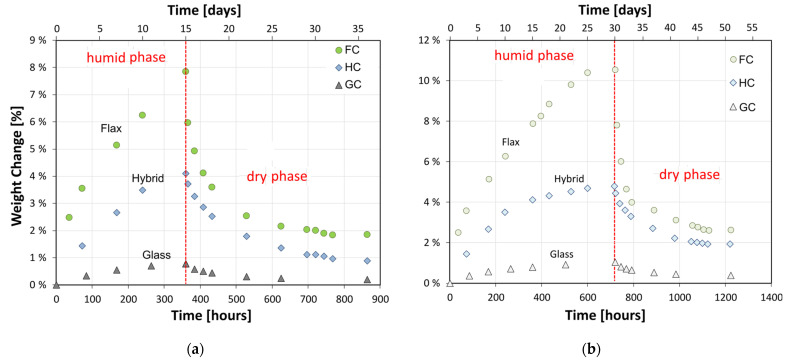
Weight change trends of composites exposed to the salt-fog for (**a**) 15 days and (**b**) 30 days.

The results for the composites exposed to the salt-fog for 15 and 30 days are summarized in Figure 1a,b, respectively.

During the humid phase, all the composites exhibited progressive weight increases at increasing time of exposure to the humid environment. This effect is more evident for the full flax reinforced composite (FC); i.e., it is gradually less relevant with the glass fiber content in the stacking sequence.

Already after 15 days of exposure to salt-fog, FC laminate is characterized by an average weight gain of 7.9%, i.e., about 10 times higher than the glass one. These results are in agreement with those of Assarar et al. [19], who demonstrated a water absorption of flax fiber reinforced composites about 12 times higher than the glass fiber one. On the other hand, the hybrid stacking sequence allowed the laminate to exhibit an intermediate behavior reaching a maximum water uptake of about 4%.

At longer salt-fog exposure times (i.e., 30 days), the water uptake trends show a progressive stabilization achieving (mainly for hybrid and glass fiber composite laminates) asymptotic equilibrium values. In particular, FC, HC, and GC laminates exhibited maximum water uptake values equal to 10.5%, 4.8%, and 1% after 30 days of exposure to the salt-fog environment, respectively.

It is widely known that flax is a porous structured lignocellulosic fiber characterized by a hierarchical structure, composed by two-layer shells and a central porous core, called lumen, thus exhibiting marked hydrophilic behavior. On the other hand, glass fibers are hydrophobic [20,21], as confirmed by glass-based laminates that show quite stable behavior in terms of weight change, compared to the other samples.

**Figure 2:**
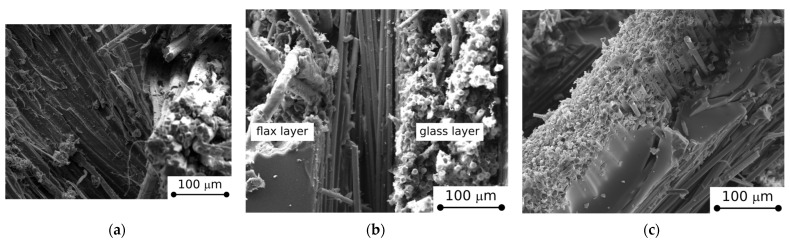
SEM micrographs of the flexural fractured surfaces of (**a**) flax, (**b**) hybrid, and (**c**) glass composites at the end of the humid/dry cycle.

As predicted, a reverse trend can be observed during the dry phase; i.e., the composites’ weight progressively decreases as a consequence of the evaporation of the previously absorbed water. This effect is more relevant in the early stages of the dry phase, and then the curve slope gradually decreases with increasing dry time, until it stabilizes at the end of the cycle. This behavior implies that the water absorbed during the humid phase can be reversibly released in a short drying period. However, by considering that a residual weight gain persists even after long drying times, some local degradative phenomena took place during the humid phase [22,23]. In particular, these phenomena are more pronounced the higher the flax content in the stacking sequence. Furthermore, the residual weight gains shown by glass, hybrid, and flax fiber reinforced composites at the end of the humid/dry cycle are in accordance with the last finding (i.e., 0.38%, 1.67%, and 2.62%, respectively). These considerations are confirmed by observing the micrographic details of the fracture surfaces of the composites (Figure 2). In particular, Figure 2a shows that the FC laminate is characterized by large fiber-matrix debonding as well as some sort of fibrillation inside the flax bundle. This effect, triggered by high water sorption experienced during the humid phase, is also favored by the weak interfacial adhesion between the natural fiber and the surrounding epoxy matrix. On the contrary, the GC sample (Figure 2c) exhibits a more stable fiber/matrix interaction. Indeed, the fiber strand is quite regular without any evidence of relevant degradative phenomena. There is a large crack in the matrix, generated during the sample fracture, probably due to interlaminar shear stresses (i.e., the crack is generated along the separation plane between two glass laminae). Finally, the HC sample (Figure 2b) shows intermediate behavior compared to the other two samples. The external glass layer appears quite homogenous with some local voids or debonding. However, these last are justified by considering the direct exposure of the external layer to the aggressive environment. On the other hand, the internal flax layer still shows quite good fiber/matrix bonding. However, the strand is clearly frayed due to the scarce interface adhesion. This also could be an indication of relevant interlaminar stresses in the plane of separation between the two layers, characterized by different stiffness, that could favor premature fractures along this plane.

To better clarify the relationship between the water absorption and the degradative phenomena, the bulk density and void content of the investigated composites were monitored at different times of the humid and dry phases (see Table 2).

The bulk density is clearly influenced by the stacking sequence of the composites; as expected, it progressively increases with the glass fiber content. In particular, the average density values of unaged samples (i.e., H0D0) are equal to 1.080 g/cm^3^, 1.364 g/cm^3^, and 1.585 g/cm^3^ for flax, hybrid, and glass composites, respectively.

Furthermore, the density of the composites increases during the exposure to the salt-fog, regardless of the stacking sequence. Nevertheless, this effect is more evident for composites containing flax fibers which, after 30 days of salt-fog exposure, show density increases equal to 3.7% (i.e., FC-H30D0 batch) and 4.4% (i.e., HC-H30D0 batch) in comparison to H0D0 batches, respectively. In other words, FC and HC aged laminates present higher density variation from the unaged status in comparison to glass ones. This means that the presence of lignocellulosic fiber in the stacking sequence favors the activation of larger degradative phenomena during the humid phase (i.e., salt-fog exposure) in comparison to the full glass composite.

This behavior can be traced to the high hydrophilicity of flax fibers, which significantly contributes to the water diffusion through the composite laminate [24]. At the same time, the weak adhesion with the hydrophobic epoxy matrix further speeds up this phenomenon at the interface area [25]. These statements can be confirmed by analyzing the void content evolution; i.e., it was found that the greater the reduction, the higher the increase in density. This indicates that the absorbed water penetrates inside the composite structure, thus reducing its empty voids [26,27]. 

Afterwards, the density of the investigated composites decreases and their void content increases during the dry phase, regardless of the stacking sequence. This probably can be ascribed to the evaporation of the water previously absorbed, which leaves empty voids or cracks within the composite [15,28]. For instance, at the end of the humid/dry cycle (i.e., H30D21 batches), flax, hybrid, and glass samples present density values equal to 1.060 g/cm^3^, 1.381 g/cm^3^, and 1.580 g/cm^3^, respectively.

Overall, the hybrid stacking sequence mitigated the effect of the hydrophilic character shown by flax fibers, thanks to the presence of outer glass layers; i.e., hybrid composites demonstrated intermediate behavior in terms of water absorption and desorption among the investigated composites exposed to the humid/dry cycle.

### 3.2. Flexural Performance Evolution

With the aim to deepen the effect of the stacking sequence on the mechanical stability of the investigated composites, it is of the utmost importance to furnish an insight into the evolution of their stress–strain curves during the humid/dry cycle.

**Figure 3:**
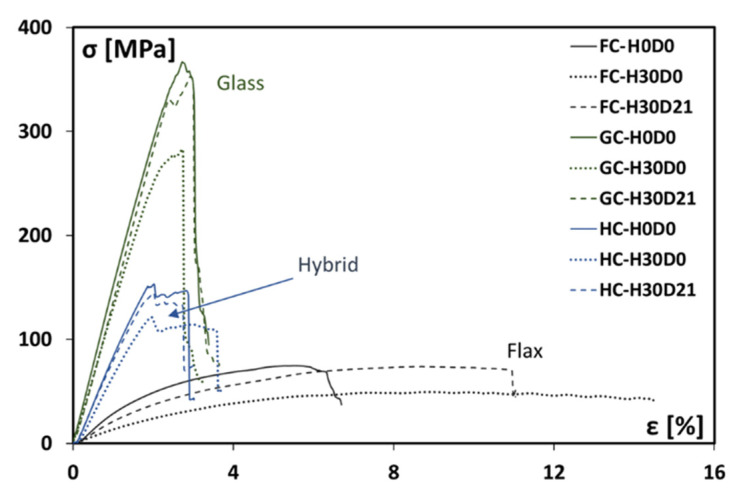
Stress–strain curves for flax (FC), hybrid (HC), and glass (GC) fiber reinforced composites at different humid and dry conditions.

First of all, Figure 3 shows the comparison between the stress–strain curves of the unaged composites (FC-H0D0, GC-H0D0, and HC-H0D0, represented by solid lines). As expected, FC laminates are characterized by the lowest strength and stiffness values among the investigated materials. On the other hand, both properties increase with the glass content; i.e., the hybrid stacking sequence allows achieving an intermediate behavior between the other batches, although it can be noted that its stress–strain trend is closer to that of the GC laminate. This is attributable to the presence in the hybrid stacking sequence of outer glass fiber layers, which offer a better reinforcing action to the applied bending stresses than flax ones. At the same time, the proposed hybrid stacking sequence is suitable to enhance the durability of the composite in severe environmental conditions thanks to the possible shielding action offered by the external layers [29].

By observing the stress–strain curves of the investigated composites at varying humid and drying times in Figure 3, it can be pointed out that all the investigated composites exhibit a reduction of their mechanical performance due to exposure to salt-fog. This detrimental effect is more marked for the FC laminate, which evidences relevant reductions both in flexural strength (i.e., identified by the maximum stress values of the curve) and stiffness (i.e., related to the initial slope of the curve) after 30 days of salt-fog exposure (i.e., −31% and −64% in comparison to FC-H0D0, respectively). In addition, a slight increase of the deformation at break is also experienced. This behavior can be ascribed mainly to the softening phenomenon due to the water absorption experienced by the hydrophilic FC laminate during the humid phase [30].

On the other hand, the GC laminate shows smaller reductions of both strength and stiffness than the FC one. In particular, GC-H30D0 samples show a maximum stress decrease of about 20.4% in comparison to the unaged samples. Analogously, their average flexural modulus at the end of the humid phase is about 10.5% lower than that of the GC-H0D0 samples.

The hybrid composite exhibits instead progressive decreases in their mechanical performance during salt-fog exposure. In particular, at the end of the humid phase (i.e., 30 days of salt-fog exposition), the strength and stiffness decrease about 23.5% and 15.5%, respectively. These findings clearly highlight the affordable stability of the HC laminate in the humid environment, thanks to the effective barrier action supplied by the external glass fiber reinforced layers.

As already stated, the durability of the investigated composites exposed to the humid/dry cycle is strictly correlated to the different stabilities of their constituents (i.e., fibers and matrix) under humid conditions. In more detail, epoxy resins can be considered the best thermosetting polymers in terms of aging resistance in seawater; i.e., their choice allows the service life of composite components in such a hostile environment to be extended [31].

With regard to reinforcement, flax fibers, being hydrophilic, are very easy to degrade and swell in such an environment, thus, in turn, favoring interfacial debonding with detrimental consequences on the overall performance of the composites [32]. On the other hand, although glass fibers also suffer physical damage and/or chemical degradation when exposed to seawater [33], they are able to guarantee better resistance to water and sodium chloride attacks in comparison to flax fibers [34,35].

The different behavior shown by flax and glass fibers clearly influences the modification induced by the humid/dry cycle on the stress–strain curves of the investigated composites.

In particular, the aged composites show an evident recovery of their mechanical performance during storage in the controlled atmosphere. All the batches show relevant increases in strength and stiffness during the dry phase; i.e., these samples (i.e., H30D21) present at the end of the humid/dry cycle mechanical properties very close to the unaged ones (i.e., H0D0). This effect is very relevant for composites having glass fibers in the stacking sequence (i.e., GC and HC laminates). On the other hand, although the FC laminate is also able to regain its initial flexural performance at the end of the humid/dry cycle, it still preserves a relevant residual plasticity, evidenced by the high deformation at break shown by the FC-H30D21 samples, i.e., about 60% higher than the unaged ones (i.e., FC-H0D0).

As well as for water adsorption and desorption, even the evolution of the mechanical performance of the investigated composites during the humid/dry cycle is greatly influenced by their stacking sequence. In particular, the presence of the outer hydrophobic glass layers, acting as protective shield, allows to preserve from the degradative phenomena the inner flax fiber reinforced layers, thus making such a stacking sequence the best compromise in terms of overall flexural properties, weight, cost, sustainability, and aging resistance [36,37].

**Figure 4:**
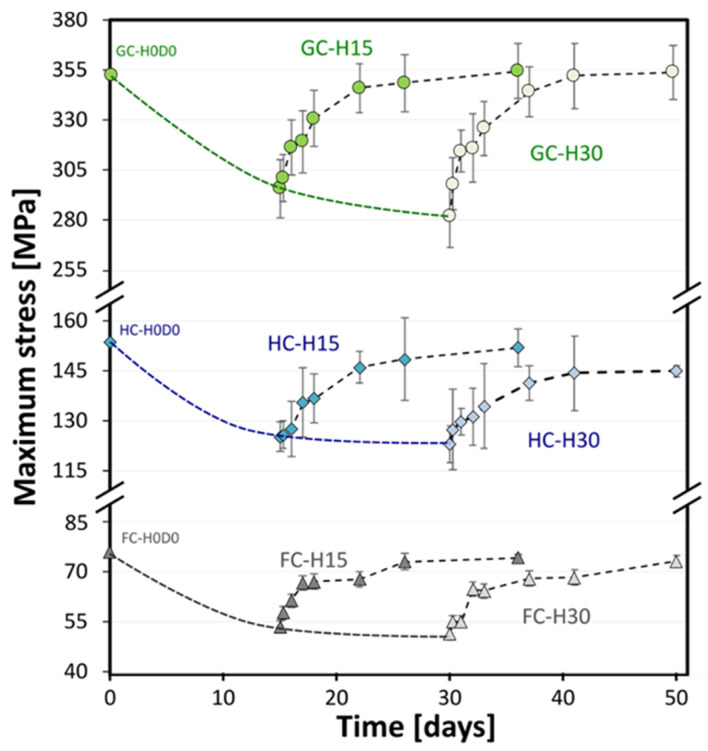
Flexural strength versus time trends for flax (FC), hybrid (HC), and glass (GC) fiber reinforced composites.

By plotting the flexural strength (i.e., the maximum flexural stress in the stress–strain curves for each aging condition) as a function of the aging time, it is possible to identify that all the investigated composites show monotone trends during the dry phase, with an increasing evolution that progressively tends toward an asymptotic value for long times (Figure 4).

As predicted, the batches exposed to salt-fog for 15 days (i.e., H15Dx) show strength values higher than those aged in the same conditions for 30 days (i.e., H30Dx), regardless of the stacking sequence. Even more interesting is the influence of the stacking sequence on the ability to regain the mechanical performance shown by the composites during the dry phase. Indeed, it is possible to notice that the GC laminate demonstrates an almost total recovery of its initial strength value (i.e., GC-H0D0, unaged samples) at the end of the humid/dry cycle, regardless of the humid phase duration. In more detail, the strength average value of the GC-H30D21 samples (i.e., initially exposed to salt-fog for 30 days and then stored in the climate-controlled room for 21 days) is equal to 353.6 MPa.

The presence of flax fibers in the stacking sequence drastically reduces the flexural strength of the unaged composites (i.e., equal to 74.5 MPa and 152.4 MPa for FC-H0D0 and HC-H0D0 batches, respectively), but it also induces more extensive degradative phenomena in the laminate during the aging cycle. In particular, the FC laminate experiences a flexural strength decrease of about 31.4% due to salt-fog exposure for 30 days, even if this weakening effect progressively decreases while increasing the dry time. This effect is more evident for the FC and HC batches. In particular, it can be observed that, by considering the samples aged for 30 days (i.e., H30D21), the HC and FC laminates reach a maximum flexural stress almost comparable to the unaged one (i.e., 9.8% and 0.2% lower than that of the unaged HC and FC samples, respectively).

**Figure 5:**
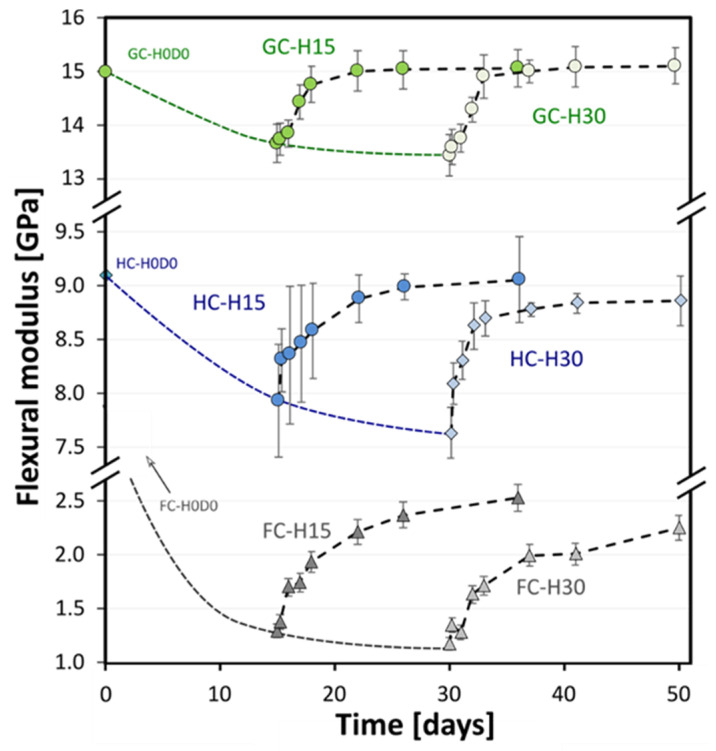
Flexural modulus versus time trends for flax (FC), hybrid (HC), and glass (GC) fiber reinforced composites.

Different considerations can arise from the comparison of flexural modulus versus time trends of all the investigated composites during the humid/dry cycle (Figure 5).

It is worth noting that the stacking sequence has a key role in the stiffness stability of composites exposed to severe environmental conditions such as salt spray. In particular, it is noted that already after 15 days of exposure in this environment, the FC-H15D0 samples showed a reduction in their modulus of about 60%. Moreover, the stiffness recovery due to the dry phase is not so effective for this stacking sequence: i.e., the residual stiffness of this composite after 21 days of drying (i.e., FC-H15D21 batch) is about 22% lower than the unaged one. This means that these environmental conditions greatly degrade the flax fiber used as reinforcement of the FC laminate, also leading to fiber softening, swelling, and weakening of the fiber–matrix interfacial bond [38,39]. In more detail, the swelling of flax fibers due to water absorption generates stress on the surrounding matrix, thus leading to microcrack formation [32]. Furthermore, the following drying causes the desorption of the absorbed water, thus resulting in fiber shrinkage and, as a consequence, fiber/matrix debonding [40], which leads to the reduction in the mechanical properties and dimensional stability of the composites [41].

On the other hand, the use of glass fibers as reinforcement of the external laminae in the stacking sequence of HC and GC laminates greatly improves their degradation resistance. Indeed, both the HC-H30D21 and GC-H30D21 batches show flexural modulus values comparable to the unaged ones (i.e., −0.5% and +0.5%, respectively). This is ascribed mainly to the hydrophobic character of glass fibers, which, in turn, evidence higher aging resistance in humid or wet environmental conditions than lignocellulosic fibers such as flax ones [19].

To better assess the performance recovery shown by the investigated composites during the dry phase of the cycle, two performance indices are defined as follows:

(1) $$FSR=\frac{\sigma_{i}}{\sigma_{0}}\cdot 100$$

(2) $$EMR=\frac{E_{i}}{E_{0}}\cdot 100$$

where the subscript 0 refers to the unaged condition (i.e., *σ*_0_ and *E*_0_ are the flexural strength and modulus average values shown by the H0D0 unaged batches, respectively), whereas the subscript *i* is related to the flexural performance shown by the investigated composites at *i*-th dry time. FSR and EMR indices are defined as the flexural strength and the flexural modulus ratio, respectively. Hence, these indices measure the mechanical performance recovery (in percentage) shown by composites during the dry phase. For instance, an FSR index equal to 80 indicates that, after a specific dry time, the aged composite exhibits a flexural strength equal to 80% of its unaged value, i.e., indicating a 20% residual strength decay. Similar consideration can be achieved for the EMR index, related to the elastic modulus recovery.

**Figure 6:**
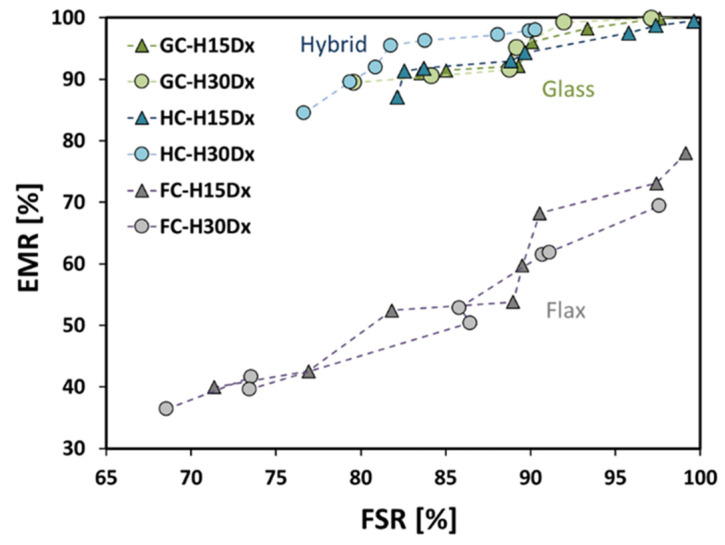
Flexural modulus index versus flexural strength index at varying drying times.

Figure 6 shows the relationship between these indexes (i.e., EMR and FSR on *y*-axis and *x*-axis, respectively) for all the investigated composites. 

First of all, it is worth of noting that flax composites evidence higher degradation induced by exposure to salt-fog (i.e., humid phase) as well as a reduced recovery of performance during the dry phase in comparison to other batches (i.e., GC and HC). This effect is particularly evident by analyzing the EMR trend of FC composites, which is at least 15% lower than the other composites (i.e., GC and HC).

The EMR vs. FSR trends for HC and GC laminates are quite comparable, thus confirming the effectiveness of hybridization on the performance stability and durability of natural-based composites. Indeed, the presence of external layers reinforced with glass fibers acting as a protection shield of the laminate “core” constituted by flax fiber reinforced layers has a beneficial impact on the stabilization of the composites’ performance under critical and variable environmental conditions.

**Figure 7:**
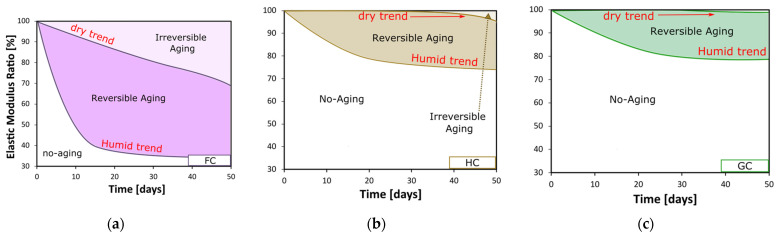
Topological map of reversible and irreversible aging of elastic modulus ratio for (**a**) flax, (**b**) hybrid, and (**c**) glass fiber reinforced composites.

To graphically support the evaluation of the degradation level of the investigated composites (i.e., at varying the stacking sequence), topological maps of the elastic modulus index (EMR) are schematized in Figure 7. These graphs allow understanding of how the degradation level can be reversibly or irreversibly recovered during the dry phase of the cycle.

In each graph, the colored area represents the region related to the aged state. This area is in turn divided into two subareas, able to discriminate the irreversible aging region from the reversible one. In particular, the reversible aging region is separated from the no-aging area by a lower line (i.e., humid trend line), which is determined from interpolation of the EMR values shown by the investigated composites at the end of the humid phase. Similarly, the interpolation of the EMR values at the end of the dry phase allows for drawing of the upper line (i.e., dry trend line). It is worth noting that the colored aging area is the widest for the FC laminate; i.e., it extends over a large region of the graph, meaning that this composite experiences significant stiffness variations due to the exposure to the humid environment. On the other hand, this area is remarkably smaller and located on the top right side of the plot in the case of the GC laminate. This finding is indicative of the relevant stability under humid conditions shown by the composite reinforced with only glass fibers.

Furthermore, the topological maps show the recovery envelope curve correlated to the progressive performance recovery of the investigated composites during the dry phase. As already stated, this curve divides the aging area into two sub-zones:

1. Reversible aging region. This refers to the fraction of performance lost during the initial humid phase, which then can be recovered by removing the adverse environmental conditions. The region is therefore associated with those degradative phenomena that can weaken the composite but in a reversible way. For instance, a possible reversible phenomenon consists in the adsorption of water in the bulk of the composite constituents (that exhibit an elastoplastic mechanical behavior in the composite laminate [18]). This absorbed water can be removed thanks to its natural evaporation during the dry phase [42];
2. Irreversible aging region. All the degradative phenomena whose effects persist once composites are removed from the aggressive environment (i.e., salt-fog exposure in this work) even after long dry times are identified as irreversible ones. Delamination, debonding, or matrix microcracks can be considered defects due to this kind of degradative phenomena. In other words, at the end of the aging cycle (i.e., salt-fog exposure followed by drying in controlled conditions), composites still maintain these defects, which physically induce a structural discontinuity in the material, thus irreversibly reducing their mechanical response.

Finally, Figure 7 shows that, concerning the composites’ stiffness, the extension of the irreversible aging region decreases with the glass fiber content in the stacking sequence; i.e., this zone is very small for hybrid and glass composites, meaning that the fraction of flexural stiffness irreversibly lost during the initial humid phase is low for these stacking sequences. This is further proof that the hybridization of natural fibers with glass fibers represents a suitable approach to stabilize the durability of NFRCs exposed to the discontinuous humid conditions typical of marine applications.

### 3.3. Performance Recovery Modeling

To deepen the performance recovery ability shown by the investigated composites, also correlating this peculiarity to their stacking sequence, an interpretative analytical model of the kinetics of the process is proposed in this work. In this way, it may be possible to define the main key features of the recovery phenomena for all the investigated composites.

**Figure 8:**
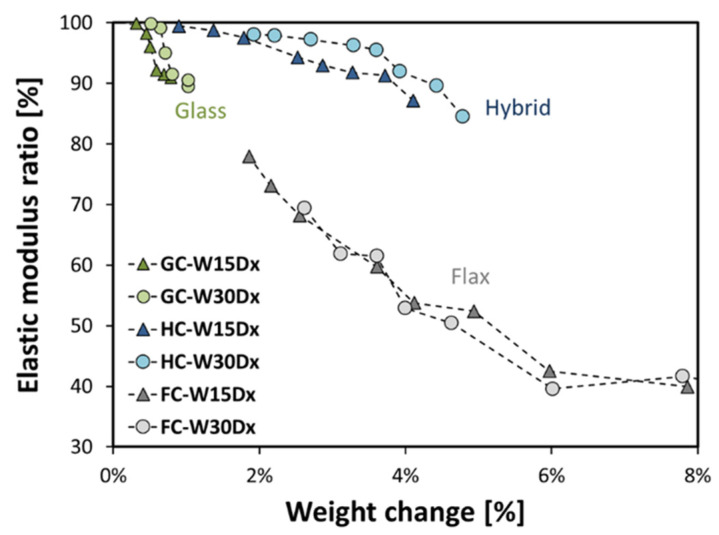
Elastic modulus ratio versus weight change for all the composite batches during the dry phase.

It is widely known that the degradative phenomena correlated to chemo-physical processes such as plasticization or the fiber–matrix interface weakening can promote significant changes in the mechanical properties of composites aged in humid conditions. Moreover, these detrimental effects can be correlated to the weight change due to water absorption/desorption experienced by the investigated composites [43,44]. With the aim of validating this statement, a correlation between the flexural strength variation and the weight change (i.e., indirectly related to water vapor evaporation) shown by composites during the dry phase was first assessed (Figure 8).

First of all, a direct proportionality between the elastic modulus ratio and weight change can be observed, regardless of the stacking sequence. To increase the readability of the plot, the weight change was limited to up to 8%. The flexural strength variation shows the same trend, not reported here for the sake of brevity. As predicted, a higher increase of weight change as well as a lower reduction in the mechanical properties occur as the natural fiber content in the stacking sequence increases.

Moreover, the water release during the dry phase is associated with a progressive desorption process. Therefore, the performance recovery of composites dried after aging in a humid environment can be ascribed mainly to the desorption phenomenon of water vapor from the material bulk. In this regard, a pseudo-second-order model already has been applied with effective results by other authors [45,46]. Hence, a performance recovery model is developed here on the basis of the pseudo-second-order model equation [47]:

(3) $$\frac{dX_{t}}{dt}={K_{2}\left(X_{e}-X_{t}\right)}^{2}$$

where *X_t_* is the performance recovery [MPa] related to the strength or modulus evolution at dry time *t*. By integration of Equation (3) at boundary conditions (*X_t_* = 0 at *t* = 0 and *X_t_* = *X_t_* at *t* = *t*), the pseudo-second-order kinetics may be expressed in a linear form as follows:

(4) $$\frac{t}{X_{t}}=\frac{1}{K_{2}X_{e}^{2}}+\frac{t}{X_{e}}$$

where *K*_2_ [MPa^−1^·mh^−1^] is its rate constant. The slope and intercept of linear *t*/*X_t_* vs. time plot can be used to determine the second-order rate constant K_2_ and the maximum equilibrium performance recovery *X_e_*, respectively. 

Furthermore, the initial performance recovery rates *h*_0,2_ [MPa·h^−1^] and half performance recovery time *h* can be calculated as:

(5) $$h_{0,2}=K_{2}X_{e}^{2}$$

(6) $$t_{1/2}=\frac{1}{K_{2}\cdot X_{e}}$$

By considering that from Equation (3), dq_t_/dt approaches $kq_{e}^{2}$ when qt→0.

Based on the previous built model, the correlation between the performance recovery and the dry time can be described by the fitting parameters summarized in Table 3.

With regard to the composite samples exposed for 15 days to salt-fog (i.e., H15 batches), the dispersion of the data does not allow for identifying a univocal trend among the three different batches. This is probably attributable to the limited aging time, which does not meaningfully differentiate the performance decay and recovery behavior among the three investigated composite batches. Different considerations can be argued considering the results referred to the H30 batches. The maximum equilibrium performance recovery *X_e_* is significantly higher for the HC and GC batches compared to the FC one for both flexural strength and modulus. This finding can be ascribed to the marked improved mechanical performance shown by the glass-based composites (i.e., HC and GC) compared to the flax-based one. Similarly, interesting information can be obtained by comparing the trends of the initial performance recovery rate (*h*_0,2_ parameter) of flax and glass fiber reinforced composites. In particular, it was found that glass fibers allow the composite to reach an initial performance recovery kinetics about 10 times higher than flax ones.

The presence of glass fibers in the stacking sequence has an effect on the desorption process and, consequently, on the recovery of the reversible fraction of the decayed mechanical performance. In more detail, glass fibers perform a shielding action against the diffusion of water through the material. Therefore, the degradation phenomena in these composite laminates are very limited, thus allowing an easier and faster recovery of performance during the dry phase.

Conversely, a greater degradation of the composite occurs in the case of FC batches. Long-term exposure in a salt-spray chamber can lead to the formation of delamination, debonding voids, or microcracks in natural fiber reinforced composites, thus exhibiting a performance recovery delay during the following dry phase.

Finally, based on the experimental results and the proposed model, a simplified approach aiming to forecast the mechanical properties of the composite subjected to a humid/dry cycle is proposed.

Considering *P*_0_ the mechanical property (i.e., flexural strength or flexural modulus) value of the unaged composite, a performance decay during the humid phase and a partial recovery during the dry phase are noted. Consequently, it is possible to identify, *Pt* = $P_{\left(th+td\right)}$, the mechanical performance at time t, as:

(7) $$P_{\left(th+td\right)}=P_{0}\frac{\alpha_{h}(t_{h})}{\alpha_{d}(t_{d})}$$

where $\alpha_{h}(t_{h})$ is defined as the decay factor related to the effect of the humid environment, i.e., the exposure to salt-fog for *t_h_* time. Analogously, $\alpha_{d}(t_{d})$ is the recovery factor due to the dry environment, referred to the drying time, *t_d_*. Both indices are lower than 1.

(8) $$\alpha_{h}(t_{h})=\frac{P_{h}@t_{h}}{P_{0}}$$

(9) $$\alpha_{d}(t_{d})=\frac{P_{h}}{P_{h}@t_{h}+X_{d}@t_{d}}$$

$P_{h}@t_{h}$ was experimentally fitted by considering that the degradation variable is a function of the water content in the composite material [48], according to the following expression:

(10) $$P_{h}@t_{h}=\left(1-\frac{P_{0}-P_{h\infty }}{P_{0}}\frac{{WU}_{t_{h}}}{{WU}_{\infty }}\right)P_{0}$$

where $P_{h\infty }$ is the degraded performance at saturation, whereas ${WU}_{t_{h}}$ and ${WU}_{\infty }$ are the water uptake values shown at time t_h_ and at saturation, respectively. According to Equation (4), *X_d_@t_d_* is defined as:

(11) $$X_{d}@t_{d}=K_{2}-\frac{K_{2}}{1+X_{e}{\cdot K}_{2}{\cdot t}_{d}}$$

**Figure 9:**
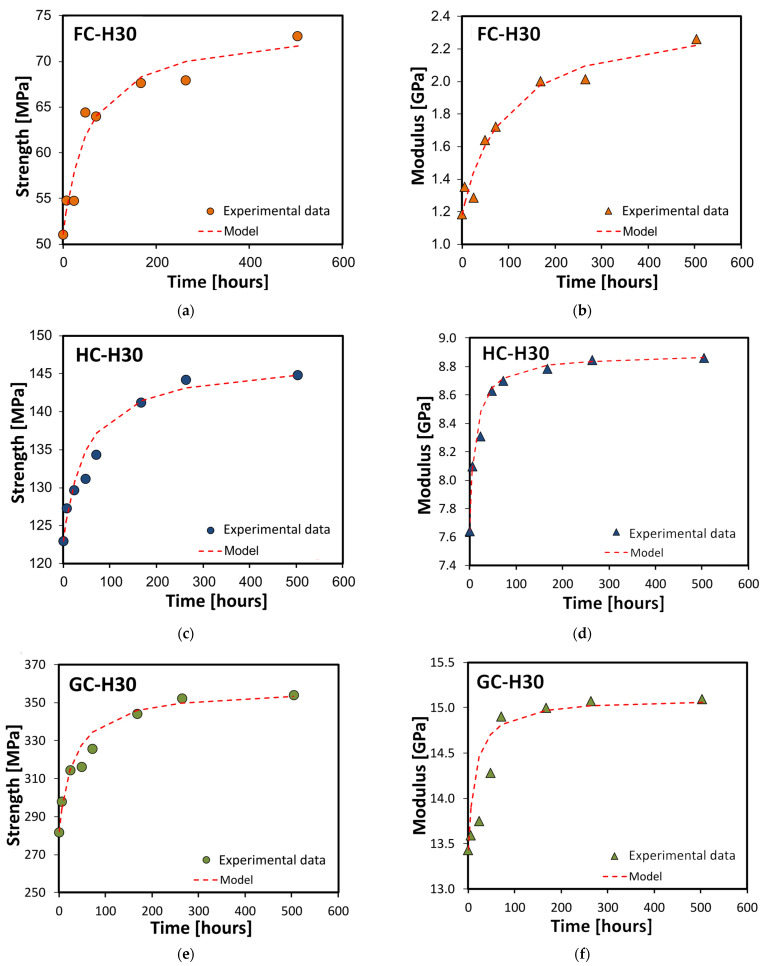
Experimental data and model fitting results referred to (**a**,**c**,**e**) flexural strength and (**b**,**d**,**f**) modulus for flax (FC), hybrid (HC), and glass (GC) composites during dry phase after 30 days of salt-fog exposure.

Figure 9 compares the experimental data and model fitting results referred to flexural strength (a, c, e) and modulus (b, d, f) for the FC, HC, and GC composites during the dry phase after 30 days of salt-fog exposure.

By observing the above graphs, it can be concluded that the simplified forecasting model suitably fits the experimental data shown by the composites during the whole dry period as regards both flexural strength and stiffness, regardless of the humid phase duration. Furthermore, the quality of the fitting was quantified using the root mean squared error [49]. In particular, the mean squared error (MSE) between the predicted values (i.e., fit measure) and the experimental data was evaluated by the R-squared value (R^2^ or the coefficient of determination). An average R² value of about 0.95 regardless of the stacking sequence was observed, indicating the good reliability of the proposed pseudo-second-order model in terms of fitting accuracy.

## 4. Conclusions

In this paper, the behavior of flax, glass, and flax–glass fiber reinforced epoxy composites exposed to a salt-fog/dry aging cycle was compared in terms of water sorption and flexural properties. To this aim, all the investigated composites were first exposed to salt-fog spray conditions for 15 or 30 days and then stored in a climate-controlled room (50% ± 10% R.H. and 23 °C ± 2 °C) for up to 21 days. 

Furthermore, a simplified theoretical model based on water uptake and a pseudo-second-order equation was proposed in order to predict both the reduction and the recovery of mechanical performances shown by composites during the humid and dry phases of the aging cycle, respectively. The forecasting model was experimentally validated and the quality of the fitting was quantified using the root mean squared error. The main findings can be summarized as follows:

- The water adsorption and desorption properties as well as the evolution of the mechanical performance shown by the composites during the aging cycle are greatly influenced by their stacking sequence. All the investigated composites exhibited an initial reduction and a subsequent recovery of their mechanical performance due to salt-fog exposure and storage in a climate-controlled room, respectively. Both the above effects are more marked with glass fiber content in the stacking sequence;
- The hybrid stacking sequence allowed achieving an intermediate behavior between the other batches, closer to that of the full glass composite. Indeed, the hybridization of inner flax reinforced layers with outer glass ones hindered the composites’ degradation due to the humid phase, also promoting their performance recovery during the dry phase;
- The hybrid stacking sequence is suitable to enhance the durability of composites exposed to aggressive environments thanks to the shielding role played by the external layers. This represents a proper compromise between flexural properties, weight, cost, sustainability, and aging resistance;
- The proposed forecasting model showed a good agreement with the experimental data during the entire aging cycle; i.e., the average coefficient of determination R² was about 0.95, regardless of the stacking sequence. This demonstrated the good reliability of the proposed pseudo-second-order model in terms of fitting accuracy.

The promising results of this study can surely provide valuable insights into the development of hybrid natural–synthetic composites suitable for marine applications, allowing for the design of materials with quite stable mechanical performance when exposed to aggressive conditions. Furthermore, the experimental approach and the theoretical forecasting model can contribute to the understanding of the performance recovery shown by fiber-reinforced composites after exposure to salt-fog, which is essential for structure design and maintenance during their service life in outdoor or marine environments.

## Figures and Tables

**Table 1:** List and details of the investigated composites.

CODE	Stacking Sequence ^1^	Thickness [mm]	Fiber Volume Content [%]	Void Volume Content [%]
HC	[G_2_/F_2_]_s_	3.56 ± 0.04	36.1 ± 0.6	6.1 ± 0.2
GC	[G_18_]	3.59 ± 0.08	40.0 ± 0.3	1.9 ± 0.1
FC	[F_5_]	3.35 ± 0.02	38.9 ± 1.0	10.6 ± 0.6

^1^ F = Flax fabric; G = Glass fabric.

**Table 2:** Density and void content for all composite batches at different humid and dry conditions.

	Density [g/cm^3^]			Void Content [%]		
	FC	HC	GC	FC	HC	GC
**H0D0**	1.080 ± 0.151	1.364 ± 0.008	1.585 ± 0.009	10.60 ± 0.50	6.09 ± 0.21	1.90 ± 0.11
**H15D0**	1.114 ± 0.018	1.417 ± 0.011	1.588 ± 0.011	11.57 ± 0.55	5.23 ± 0.27	1.67 ± 0.16
**H30D0**	1.120 ± 0.018	1.423 ± 0.014	1.588 ± 0.014	12.21 ± 0.44	5.34 ± 0.35	1.71 ± 0.20
**H15D21**	1.075 ± 0.015	1.373 ± 0.014	1.581 ± 0.016	12.00 ± 0.58	6.13 ± 0.34	2.11 ± 0.23
**H30D21**	1.060 ± 0.022	1.381 ± 0.014	1.580 ± 0.014	13.60 ± 0.48	6.32 ± 0.35	2.21 ± 0.20

**Table 3:** Fitting parameters based on the pseudo-second-order model.

			Flexural Strength			Flexural Modulus		
			FC	HC	GC	FC	HC	GC
**H15**	***X_e_***	MPa	21.505	38.725	63.743	1.374	1.184	1.681
	***K*_2_**	MPa^−1^·h^−1^	0.0013	0.0001	0.0003	0.0109	0.0247	0.0075
	***h*_0,2_**	MPa/h	0.633	0.195	1.250	0.020	0.035	0.0213
	***t*_1/2_**	h	0.981	0.667	0.981	0.969	0.986	0.883
**H30**	***X_e_***	MPa	22.857	23.907	75.857	1.236	1.250	1.681
	***K*_2_**	MPa^−1^·h^−1^	0.0008	0.0009	0.0004	0.0085	0.0706	0.0390
	***h*_0,2_**	MPa/h	0.420	0.489	2.368	0.013	0.110	0.110
	***t*_1/2_**	h	54.455	48.865	32.036	94.680	11.326	15.263

## Data Availability

The data presented in this study are available on request from the corresponding author.
